# Al(Salen) Metal Complexes in Stereoselective Catalysis

**DOI:** 10.3390/molecules24091716

**Published:** 2019-05-02

**Authors:** Andrea Gualandi, Francesco Calogero, Simone Potenti, Pier Giorgio Cozzi

**Affiliations:** 1 ALMA MATER STUDIORUM Università di Bologna, Dipartimento di Chimica “G. Ciamician”, Via Selmi 2, 40126 Bologna, Italy; francesco.calogero@studio.unibo.it (F.C.); simone.potenti@sns.it (S.P.); 2 Scuola Normale Superiore, Piazza dei Cavalieri 7, 56126 Pisa, Italy

**Keywords:** aluminum, aluminum metal complexes, salen, chiral ligand, Lewis Acid, catalysis, stereoselective reactions, bifunctional catalysis, organic synthesis

## Abstract

Salen ligands are a class of Schiff bases simply obtained through condensation of two molecules of a hydroxyl-substituted aryl aldehyde with an achiral or chiral diamine. The prototype salen, or *N*,*N*′-bis(salicylidene)ethylenediamine has a long history, as it was first reported in 1889, and immediately, some of its metal complexes were also described. Now, the salen ligands are a class of N,N,O,O tetradentate Schiff bases capable of coordinating many metal ions. The geometry and the stereogenic group inserted in the diamine backbone or aryl aldehyde backbone have been utilized in the past to efficiently transmit chiral information in a variety of different reactions. In this review we will summarize the important and recent achievements obtained in stereocontrolled reactions in which Al(salen) metal complexes are employed. Several other reviews devoted to the general applications and synthesis of chromium and other metal salens have already been published.

## 1. Introduction

**Figure 1:**
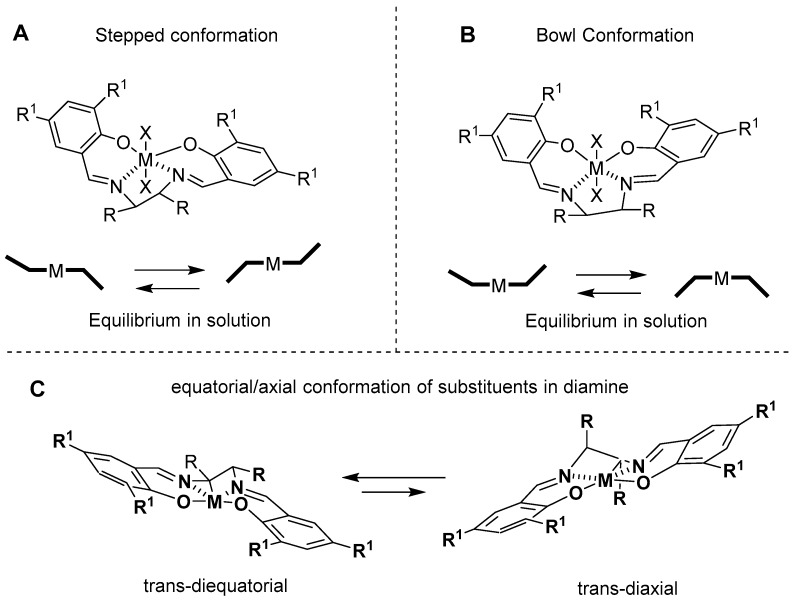
Possible conformations of M(salen) complexes.

Schiff bases are widely used as ligands in coordination chemistry and catalysis [1], and their complexes can be accessed by a variety of methodologies. As representative in the class of Schiff complexes, salen metal complexes have been explored in many applications, from material science and catalysis to coordination chemistry [2,3,4,5,6,7,8]. Salen compounds are typically prepared by a condensation reaction between aromatic *ortho*-hydroxyaldehydes with primary 1,2-diamines in a 2:1 molar ratio respectively [5]. Both chiral and achiral salen Schiff bases are obtained by these methodologies. Many different types of chiral salens, containing different stereogenic elements (centers, axes, planes), have been prepared, and salens have also been inserted in more complex structures, such as DNA [9]. The aryl residues of a salen compound have been decorated with groups able to control the stereochemical conformation of the ligand, as well as to hinder particular reaction pathways. In addition, functional groups such as Brønsted bases have also been inserted in order to introduce a bifunctional behavior of the salen metal complexes. Salen are tetradentate ligands able to coordinate a variety of metals by subtle arrangement of the imine and hydroxyl group. With large metals the coordination becomes more pyramidal, while with small metals a planar arrangement is preferred. Although the stability of metal salen complexes can be influenced by the presence of organometallic species in solution [10], and exchange between metals could be observed, normally salen complexes are quite stable and chemically inert. Depending on the metal, one or two apical coordination sites are available, although a different arrangement can be observed [11]. Salens are able to transmit chiral information in a very effective way in many catalytic transformations. The stereochemical information is transmitted by various crucial features of the salen ligand, here summarized. First, the nature of the metal and its oxidation state strongly affect the coordination. In oxo or imido salen complexes, one apical coordination site is occupied by the oxo or imido ligand respectively. What is especially important for the stereochemical outcome of the reaction is the salen metal complex conformation that is influenced by the nature of the ancillary ligands. The conformation of salen complexes in solution is important to understand the catalytic properties. Different conformations are in equilibrium. However, generally, a stable octahedral configuration, with the metal tightly coordinate to the two nitrogen and oxygen atoms, is observed by X-ray crystal structures. Chiral salen complexes adopt two different types of conformation, namely “stepped” conformations with the arrangement of atoms illustrated in Figure 1A.

**Figure 2:**
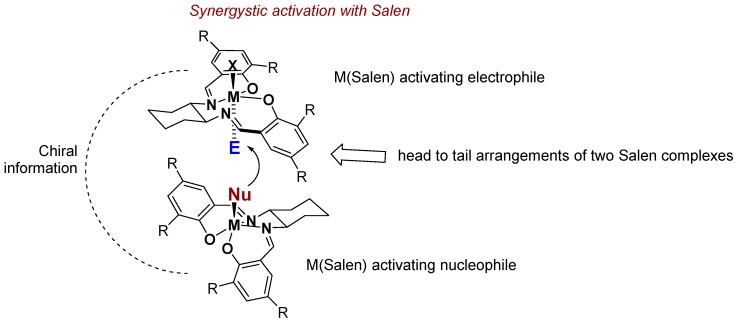
Bifunctional behavior of salen complexes.

A different type of conformation is the so-called “bowl-shaped” conformation (Figure 1B) where the structure of the complex is now arranged as an envelope shape. Whenever the diamine backbone bears stereogenic centers, there is an equilibrium between the possible stepped conformers. The substituents of the diamine moiety can be arranged in a *trans*-diaxial or *trans*-diequatorial manner (Figure 1C). The stereochemical information is therefore transmitted by these chiral conformations, and the whole salen framework hinders one possible reaction pathway. This is crucial for the effective design of chiral salen metal complexes, as their accessibility is not only obstructed by the stereocenters of the diamine. Although the diaxial conformation of the diamine substituents seems unfavored, the presence of chelating groups on the diamine can drive the equilibria towards the formation of a more stable diaxial isomer, with a concomitant coordination of the chelating groups to the apical positions. In addition, using an achiral diamine in the preparation for the salen metal complex can lead to complexes with a chiral stepped conformation. In the presence of a chiral counter ion, and using a cationic salen metal complex, one of the two chiral stepped conformations can be stabilized [12]. To add further complexity to the design of effective salen complexes for catalysis, in many reactions in which these are used, two salen metal complexes can work cooperatively. Indeed, a precise arrangement of two salen units is indispensable for activating the nucleophile and the electrophile [13]. Thus, the arrangement is crucially controlled by active conformations. Under this perspective, oligomeric salen metal complexes were purposely designed for taking advantage of the bifunctional behavior (Figure 2).

In this review, we have focused our attention on the aluminum complexes of salens, reporting the applications of the stable and effective complexes in different catalytic processes. 

## 2. Aluminum: General Properties and Character

Aluminum is the major constituent of many common minerals. Its name refers to the alums, minerals containing aluminum. Because of its availability and its capability to form alloys with peculiar characters, aluminum has found many important industrial applications. The toxicity of aluminum is considered low as compared to other metals. In aluminum complexes, it is almost present exclusively in the +3 oxidation state although interesting examples of Al(I) complexes were also reported [14]. Aluminum(III) complexes have been mainly employed in many homogeneous catalytic transformations that required a Lewis acid-type activation with polar substrates [15]. The activation is determined by the oxophilic nature and Lewis acidity of aluminum complexes. The possibility of tuning the Lewis acidity by making use of appropriate ligands—in particular salen ligands, as discussed in this review—allows to control the reactivity in many catalytic reactions. Low coordinated aluminum cationic compounds are strong Lewis acids, while a neutral five or six coordination arrangement, such as the one observed in the case of Al(salen), gave moderate electrophilic species. Although aluminum cannot change oxidation states in its complexes, they are able to promote redox reactions [15]. Free metal salts and aluminum complexes have been used to catalyze hydride transfer processes between organic molecules, the epoxidation of alkenes, and the activation of allylic and aliphatic C–H bonds [16]. In this perspective, the use of tailored ligands improved the chemo-, regio-, and/or stereoselectivity of the reactions catalyzed by Al(III) salts. The ability of Al(III) complexes to promote these reactions is due to the activation of two organic molecules in the redox chemistry. A well-known example of Al(III)-promoted redox reaction is the Meerwein-Schmidt-Ponndorf-Verley (MSPV) reduction of carbonyl compounds by primary or secondary alcohols [17]. Furthermore, the importance of the use of aluminum organometallic complexes in the polymer industry is enormous. Polyolefin production accounts for more than half of the plastics demand around the world and is still increasing. The heterogeneous conditions for the process use as principal catalytic system the MgCl_2_-supported Ziegler−Natta catalysts, composed of titanium complexes supported onto a MgCl_2_ matrix. It is important to stress the use of aluminum alkyls AlR_3_ (R = alkyl) as co-catalyst in the polymerization [18].

The photophysical behavior of Schiff-base complexes with several different metals has been previously investigated by some of us [19]. Among all the studied salen complexes, ClAl(salen) presented interesting features, showing good chemical stability, high absorption coefficients and fluorescence quantum yield. In addition, we showed that the fluorescence intensity of ClAl(salen) was modulated by the presence of coordinating species, such as carboxylate anions, allowing the association process to be monitored with high sensitivity. Photoredox catalysis is now undergoing very active investigations [20]. In particular, application of Earth-abundant metals in photoredox catalysis is an emergent topic, as the mainly used photocatalysts are based on rare and toxic ruthenium(II) and iridium(III) complexes. Photoactive complexes based on Cu(I), Zn(II), Cr(III), Co(III) and Fe(II) were reported in various photoredox reactions [21]. Aluminum is the most abundant metal in the Earth’s crust and is relatively non-toxic compared to cobalt and chromium. Therefore, the photophysical properties of Al(salen) are also worth further investigation in the photoredox catalytic applications [22].

## 3. Al(Salen): Synthesis, Properties and Coordination Chemistry

**Scheme 1:**
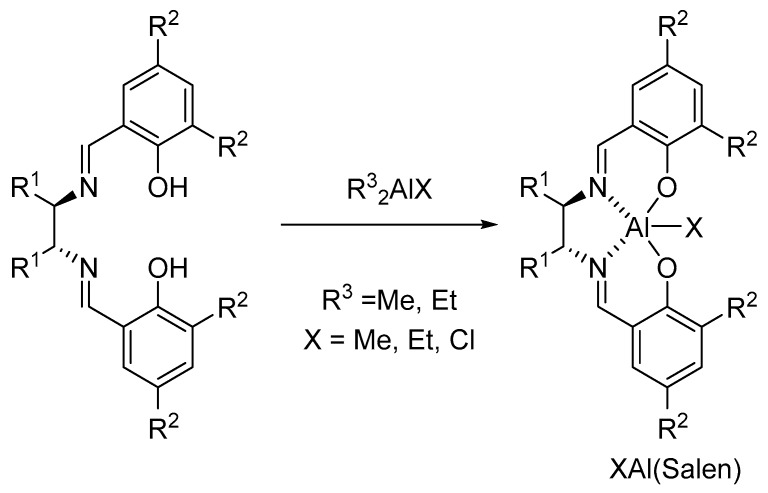
General synthesis of XAl(salen) complexes.

The preparation and chemistry of XAl(salen) species were reviewed by Atwood [23]. Al(salen) complexes are conveniently prepared by combining the chiral or achiral salen ligand with available solutions of trialkyl aluminum reagents in an aprotic solvent at room temperature (Scheme 1). Generally, the reaction is performed in toluene.

With such an approach, the desired five-coordinate alkyl derivatives of salen are obtained in very good yields [24,25]. XAl(salen) compounds, with X = Cl, can be accessed in a similar way by using dialkylaluminium chloride [26]. In these reactions, HCl can be formed as a byproduct. Therefore, the addition is generally performed at low temperatures to avoid decomposition of the aluminum salen complexes; the presence of hindered groups in the backbone of the salen can influence the yield and the solubility of the former. Generally, the complexes bearing substituted aromatic rings in the salen ligand are easily isolated after concentration of the solution. All Al(salen) complexes are monomeric, with a coordinate metal-alkyl or -halide unit. It is possible to verify the formation of the desired complex by observing the ^1^H-NMR signals. Generally, AlMe_3_ or AlEt_3_ are used for the synthesis of the Al-complexes. The signal for the methyl group linked to the aluminum can be found in the range between −1.3 and −0.98 ppm. It also is possible to record the ^27^Al-NMR. However, this opportunity is generally not often explored. The XAl(salen) compounds show a five-coordinate aluminum atom. The geometry of coordination can be trigonal bipyramidal or square pyramidal, according to the nitrogen backbone of the salen. The more rigid the framework is, the less possible it is to observe the trigonal bipyramidal geometry. The introduction of substituents in the aryl moiety does not modify the distance between the apical alkyl group and aluminum in a very large manner. Alkyl groups that occupy the apical position can be replaced by alcohols through alkane elimination [27]. It is also possible to introduce R_3_SiOH [25]. For catalytic purposes, the possibility to obtain cationic Al(salen) compounds is significant [28,29]. The reaction solvent, due to its Lewis basicity, can be able to induce the formation of cationic Al(salen)^+^Cl^−^ starting from the ClAl(salen) [29]. Notably, although this reaction can be induced by water, the Al(salen) complexes are stable in aqueous solution, and the dissociation is also possible with chiral Al(salen) complexes [30]. In water solution, two molecules of water are able to coordinate the aluminum complexes. In other reactions, acidic phenolic compounds have been used for the construction of tailored Al(salen) or Al(salophen) complexes for multiple purposes [31]. Kinetic and reaction rates of Al(salen) catalysts are determined by different factors. On the one hand, since Al(salen) behaves as Lewis acids in the case of reactions performed in coordinating solvents, these can compete with the activation of the desired substrate, slowing down the reaction rate, particularly if the rate of exchange is not high. On the other hand, Al(salen) complexes show a cooperative behavior in many catalytic reactions, as they are able to activate both the electrophile and the nucleophile in the transition state. The Al(salen) molecules bring the two reactants close in proximity. In this case, kinetics parameters are affected by this bimetallic pathway. Supporting the catalyst in polymeric matrix, favors this proximity and increas the efficiency of the process [32].

## 4. Chiral Al(salen) Complexes Used in Stereoselective Reactions

**Figure 3:**
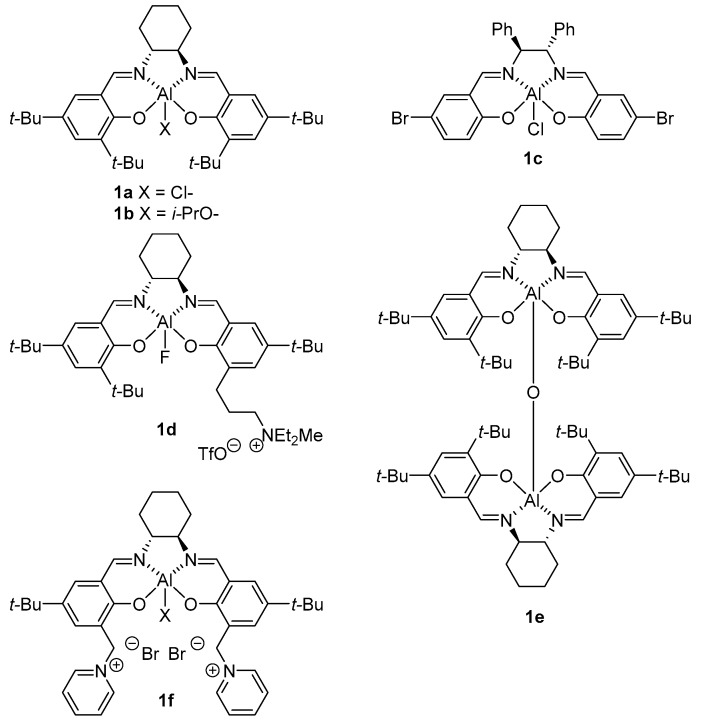
Effective Al(salen) complexes used for stereoselective reactions.

All the effective Al(salen) complexes used for the stereoselective reactions described in the review, are reported in Figure 3. Although different stereocenters were introduced in the diamine backbone, the effective design first described by Jacobsen for manganese-promoted epoxidation [33] is quite effective. In Mn(salen)-promoted epoxidation, the stereoselective reaction is due to a single unit of the salen. In many reactions in which Al(salen) acts as a Lewis acid, the trajectory of the incoming nucleophiles is effectively controlled by the cyclohexyldiamine backbone when hindered *tert*-butyl substituents are introduced in the aldehydic moieties.

## 5. Addition of CN to Electrophiles Promoted by Chiral Al(Salen) Complexes

**Scheme 2:**
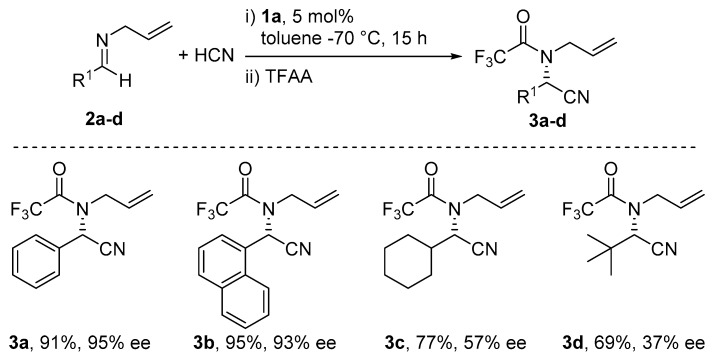
Chiral Al(III)(salen)-mediated Strecker reactions.

The Strecker reaction is one of the most important and practical ways to access amino acid derivatives [34]. The reaction consists of the addition of cyanide to imines. Many stereoselective methodologies were reported using chiral auxiliaries covalently linked to the amine moiety [35]. One of the first asymmetric methodologies for the addition of cyanide ion to imines was reported by Jacobsen using a chiral Al(III)(salen) complex (Scheme 2). [36]

Following the chemistry of the chiral Mn-, Co- and Cr-(salen) complexes studied by Jacobsen, which were proven to promote a plethora of interesting reactions, he explored the addition of cyanide ion to imines promoted by various M(salen) complexes. This investigation revealed that many salen complexes (Ti, Cr, Mn, Co, Ru) were all able to promote the reaction. ClAl(salen) **1a** was found as the best suited catalyst. Such catalyst was obtained by reacting the respective salen ligand with Et_2_AlCl in CH_2_Cl_2_/toluene mixture as the reaction solvent. A remarkable observation is related to the absence of strict anhydrous conditions in the reaction mixture, suggesting that the effective cyanating species was HCN, formed by the hydrolysis of the cyanide source Me_3_SiCN. Low temperatures were necessary to suppress the background reaction. The reaction was employed in the synthesis of a non-natural aminoacids from **2a–d**. Although the mechanistic investigation was not reported in successive studies, the reaction probably occurs via a double activation process where a CNAl(salen) attacks an Al(Salen)-coordinated imine in the enantiodetermining step.

**Scheme 3:**
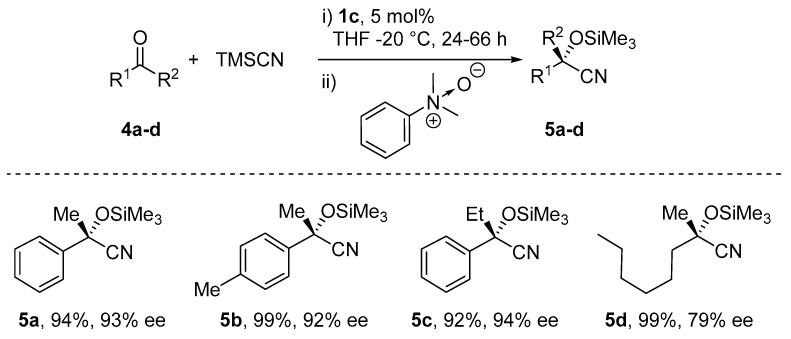
Addition of CN to imines promoted by ClAl(salen).

While Belokon and North developed effective and active Ti- and O=V-(salen) systems for the addition of Me_3_SiCN to aldehydes and ketones [37], Feng reported the use of Al(salen) in the presence of *N*,*N*-dimethylaniline *N*-oxide (DMAO) as an activating agent (Scheme 3) [38].

**Figure 4:**
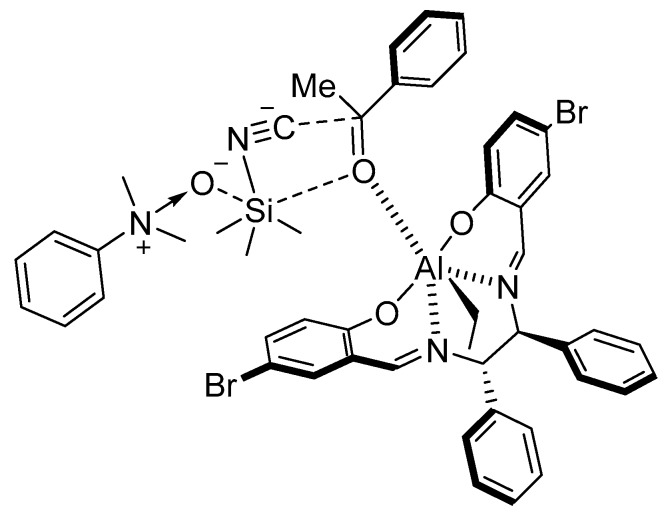
Proposed transition state for addition CN to imines.

The double activation catalysis realized by Feng was able to overcome the low electrophilicity of ketones, allowing a stereoselective addition in good yields and good ees. The catalytic system consists of a chiral Al(salen) complex and an amine *N*-oxide. It was more active for aliphatic ketones (TON 1000) and it was applicable to a wide range of aliphatic and aromatic ketones, converting them to the corresponding cyanohydrin O-TMS ethers in excellent yields and with high enantioselectivities (94% ee for aromatic ketones, 90% ee for aliphatic ones). The authors analyzed the reaction from a mechanistic point of view, showing that the Al(salen) was playing the role of Lewis acid, coordinating the ketone thanks to the free coordination site, while the N-oxide was acting as a Lewis base, activating the TMSCN (Figure 4).

**Scheme 4:**
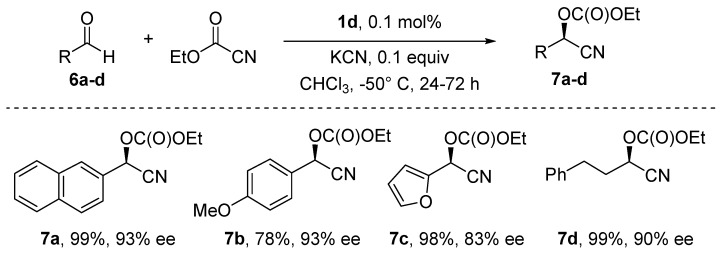
Strecker reaction reported by Peters using FAl(salen) derivatives.

Further results about such system were reported by Carpentier in 2008 [39], who has evaluated, defined and isolated Al(salen) complexes in the reaction. The isolated catalyst was able to promote the reaction more effectively. In addition, a key feature was encountered in evaluating different XAl(Salen), leading to the identification of a highly active and productive species of catalyst in which the X group was hexafluoro-2-propoxide. Zhou reported [40] a chiral (salen)Al(III) incorporating the (*R*,*R*)-11,12-diamino-9,10-dihydro-9,10-ethanoanthracene moiety as the chiral backbone. The aluminum complex was prepared through the standard reaction of the salen ligand with Et_2_AlCl and it was used as a catalyst for the stereoselective addition of TMSCN in the presence of tributylphosphine oxide as the activating agent of TMSCN. By using 1 mol% of the complex, cyanohydrins of aromatic aldehydes were isolated in 76–92% ees and in high yields (85–94%) as trimethylsilyl ethers. The reaction with alkyl aldehydes was less stereoselective as the product was isolated with up to 42% ee. In the attempt to prepare a recoverable Al(salen) complex for stereoselective catalysis, a heterogeneous Al(salen) catalyst was synthesized and covalently attached to a polystyrene resin, such as Merrifield and JandaJel resins. The solid-supported catalysts were characterized by typical solid state analytical and spectroscopic techniques, and they were tested for the Strecker type reaction. However, these showed quite modest results in the case of Al(salen) complexes [41]. Peters reported a recent improvement for the preparation of a cooperative catalyst (Scheme 4) [42,43].

With the idea to use a “naked” cyanide source and, at the same time, to use the ammonium ion to coordinate the cyanide, Peters designed the catalyst **1d** shown in Figure 2. After a survey of different Al sources, the use of Me_2_AlF gave high yield and ees. The superior performance of the bifunctional Al(salen) was ascribed to an enhanced stability and Lewis acidity. The Al-F bond is quite stable, and the catalyst did not decompose during the reaction, allowing for its subsequent recovery. Theoretical calculation performed by DFT showed that the Al-F bond in the complex is strongly polarized towards the F atom, thus leading to an Al atom with a higher partial charge compared to other Al(salen)metal complexes, such as ClAl(salen) or MeAl(salen). The Al-F bond is ionic in nature, with an enhanced capability to behave as a Lewis acid.

## 6. Michael-Type Reactions Promoted by Chiral Al(salen) Complexes

**Scheme 5:**
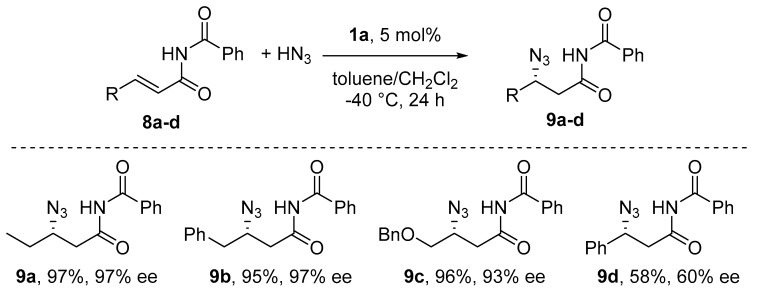
First example of the use of Al(salen) complexes in Michael additions.

The conjugate Michael reaction is one of the most powerful methods for the formation of carbon–carbon bonds in organic synthesis [44]. Both organometallic [45] and organocatalytic methodologies [46] have been significantly improved during the past years. Al(salen) metal complexes were used in promoting a series of stereoselective Michael reactions with different nucleophiles. Remarkably, the other metal salen complexes were often found ineffective in these reactions, showing the importance of the Al(salen) in these transformations. The enantioselective conjugate addition of hydrazoic acid (HN_3_) to α,β-unsaturated imides catalyzed by the Al(salen) complex was reported by Jacobsen in 1999, as the first example of the use of the Al(salen) complexes in Michael additions (Scheme 5) [47].

The reaction occurs through a bifunctional catalysis, with the Al(salen) acting as a Lewis acid activating the Michael acceptor (compound **8**, Scheme 5) and, contemporaneously, another molecule of ClAl(salen) forming the N_3_Al(salen) species. The MeAl(salen) used as a pre-catalyst reacts with HN_3_ forming an active N_3_Al(salen) metal complex. The reaction was also possible with N-alkylmaleimides, leading, under optimized conditions, to the desired azide adduct in 94% ee and 93% yield. Based on the interesting results obtained with imides, other substrates were investigated and finally *N*-benzoyl imide derivatives **8** were found to be active substrates, allowing the reaction at low temperatures. These derivatives could be easily prepared by a Horner-Emmons reaction, starting from an aldehyde and a phosphonate. Maleimides were found particularly active for the Michael reaction with Al(salen). The reaction was then studied with different imides and good results were obtained for all the substrates. Only cinnamate derivatives were considerably less reactive than the alkyl-substituted substrates. Although the reaction could be rationalized as a bifunctional catalysis, where the N_3_Al(salen) is the nucleophile, kinetic studies reported in the discussion established that the rate of the conjugate addition reaction displays a first-order dependence on Al(salen) catalyst. The kinetic data suggested that Al(salen) acts as a Lewis acid, coordinating the imide. The imide substrates were also capable of conjugate addition of carbon-based nucleophiles.

A high enantioselective conjugate additions of electron-deficient nitrile derivatives to acyclic α,β-unsaturated imides, catalyzed by a Al(salen), was reported by Jacobsen [48]. Quite remarkably, the active Al(salen) complex was found to be the μ-oxo [(*S*,*S*)-(salen)Al]_2_O dimer **1d**, that is prepared by partial hydrolysis of the MeAl(salen) [26].

**Scheme 6:**
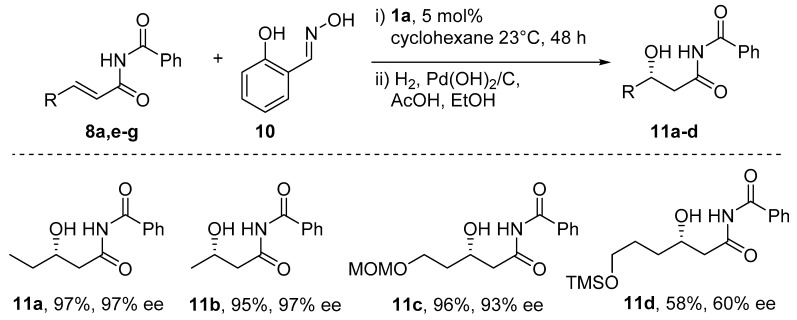
Michael addition to imide substrates reported by Jacobsen.

The scope of the reaction was quite broad with respect to the electrophile. The reaction was observed to occur better in apolar solvents, leading to increased enantioselectivities. The absolute configuration of the final products was consistent with the previous reported Al(salen) reactions. In another Michael reaction described by Jacobsen’s group, an oxygen-centered nucleophile was reported [49]. In fact, due to the reversibility of the reaction, the addition of oxygen nucleophiles to Michael acceptors was rather challenging. Jacobsen has used a masked oxygen nucleophile, the salicylaldoxime **10** (Scheme 6), for the Michael addition to imide substrates.

**Scheme 7:**
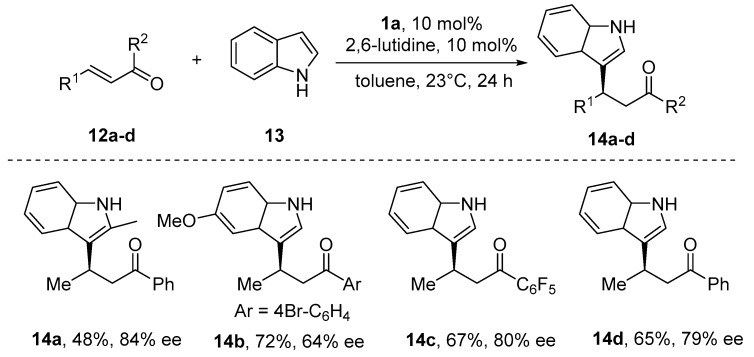
Conjugate addition of indoles as nucleophiles to ketones, in presence of Al(salen) complex.

After deprotection, the process can give a practical access to β-hydroxycarboxylic acid derivatives, with high stereoselection. Furthermore, in this case, the [(*S*,*S*)-(salen)Al]_2_O dimer was found the most active catalyst. The free OH group was obtained after hydrogenolysis of the crude oxime ethers, affording the product with *R* configuration at the newly formed stereocenter. Different imides bearing chiral alkoxy groups were also evaluated in double diastereoselective addition, affording quite high ratios for the desired diastereoisomer. To add further interest to the paper, the final transformation of the acylamides into esters was possible through a direct reaction with EtOH in the presence of catalytic amounts of Er(OTf)_3_. The effective enantioselective conjugate addition of indoles as nucleophiles to *E*-arylcrotyl ketones in the presence of an Al(salen) complex was reported by Bandini and Umani-Ronchi (Scheme 7) [50,51].

**Figure 5:**
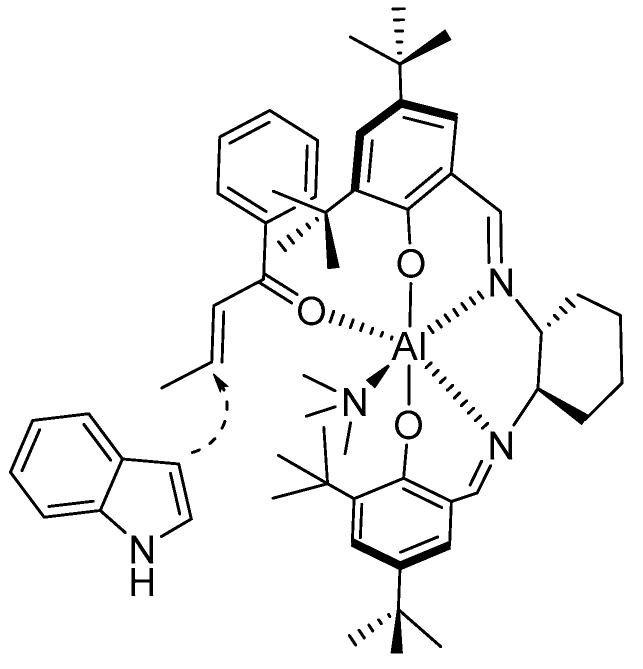
Proposed transition state for addition of indoles as nucleophiles to unsaturated ketones, in the presence of an Al(salen) complex.

In such reaction, ClAl(salen) was found to be active in the presence of a catalytic amount of coordinating base (10 mol%). The isolated products were obtained with ees up to 89% when 2,6-lutidine was utilized. For the first time in salen-mediated reactions, a theoretical investigation at the B3LYP level of theory was performed to explain the obtained results. The structural optimization of penta- and hexacoordinate complexes between the Al(salen), the base and an unsaturated ketone substrate revealed an interaction between the coordinating base and the aluminum complex. This interaction generates a stable cationic hexacoordinate chiral *trans* complex, like the one arising whenever a coordinating base such as Me_3_N is used together with an enone. This arrangement can be considered as the Lewis acid complex responsible for the stereocontrol (Figure 5).

**Scheme 8:**
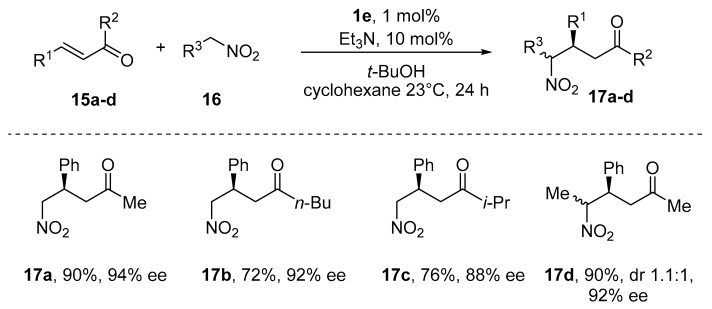
Nitro-Michael reaction promoted by a chiral Al(salen) complex.

The monomeric activation of the electrophile (i.e. the enone) was also confirmed by kinetic evidences and non-linear effect (NLE) investigations, ruling out possible double activation pathways. By using a catalytic amount of [(*S*,*S*)-(salen)Al]_2_O, it was also possible to promote the asymmetric conjugate addition of NH-heterocycles (e.g. purines, benzotriazoles) to α,β-unsaturated ketones and imides, a rather direct and useful methodology for the synthesis of non-natural nucleotides [52]. Pursuing further studies with these electrophiles, Jacobsen reported a general approach for highly enantioselective and efficient conjugate addition of carbon and nitrogen nucleophiles catalyzed by [(*S*,*S*)-(salen)Al]_2_O [53]. As was reported by Bandini and Umani-Ronchi, the simple-one point coordination of the electrophile is sufficient for observing a quite high stereoselective addition of a variety of nucleophiles (nitriles, nitroalkanes, and hydrazoic acid) (Scheme 8). The application of this methodology to the total synthesis of (+)-lactacystin was reported [54].

**Scheme 9:**
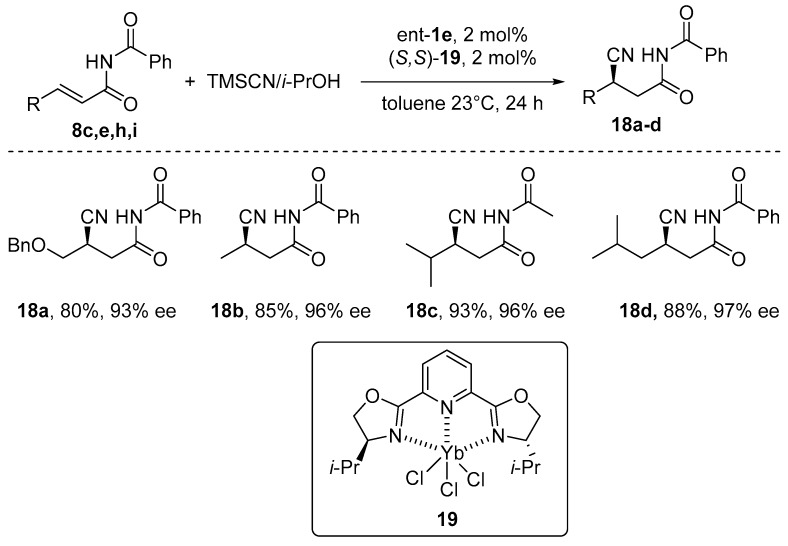
Double activated conjugate addition mediated by Al(salen) and (pybox)YbCl_3_.

In all the described Michael-type reactions, mechanistic investigations revealed that Al(salen) complexes effectively behave as Lewis acids, capable to coordinate the unsaturated compounds, controlling the approach of the nucleophiles. However, the addition of cyanide to unsaturated amides was proven unsuccessful. Regarding the capability of Al(salen) complexes to activate unsaturated imides, Jacobsen wanted to investigate the possibility to perform a double activation process, adding a complex able to activate the nucleophilic cyanide towards the addition [55]. In order to improve the rate and scope of the reaction, the incorporation of a (pybox)YbCl_3_ complex (Scheme 9) was considered.

**Figure 6:**
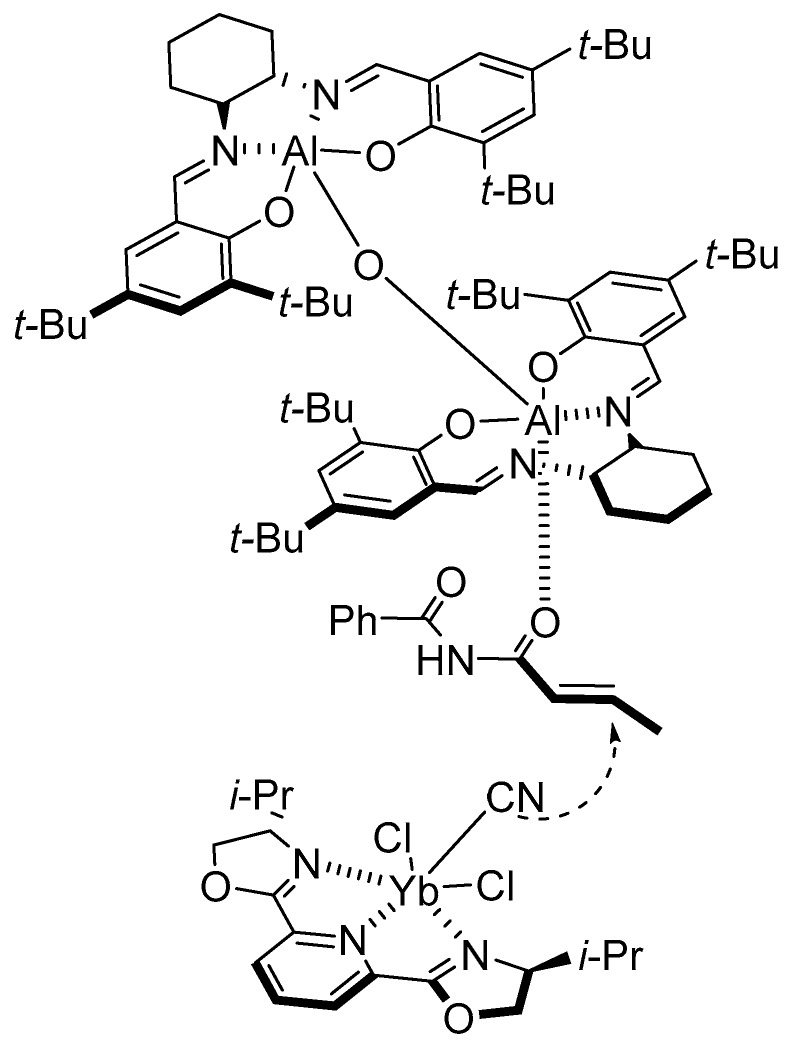
Proposed transition state in the addition of CN to activated unsaturated imides.

The combination of the two catalysts led to a highly reactive and enantioselective system. Mechanistic investigations carried out on the dual-catalyst system revealed a first-order dependence on both [(*S*,*S*)-(salen)Al]_2_O and (pybox)ytterbium (Figure 6). The chiral complex also works in matching combination, since replacing the ytterbium complex with its enantiomer led to decreased ees and conversions. Additionally, a similar active covalently-linked dinuclear [Al(salen)O]_2_–(PyBOX)ErCl_3_ complex was found relatively active in the conjugate cyanation of α,β-unsaturated imides [56].

**Scheme 10:**
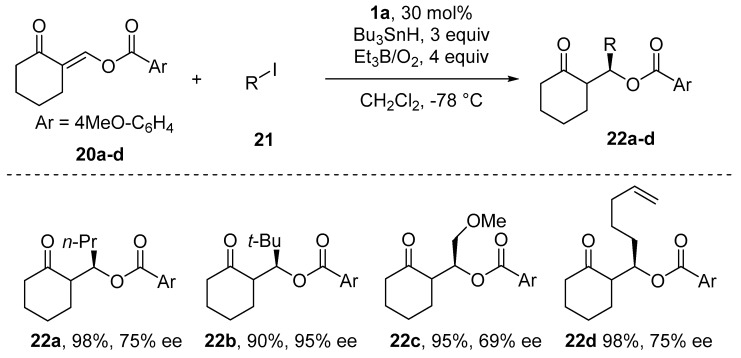
Radical addition to a cyclic ketone reported by Sibi.

**Figure 7:**
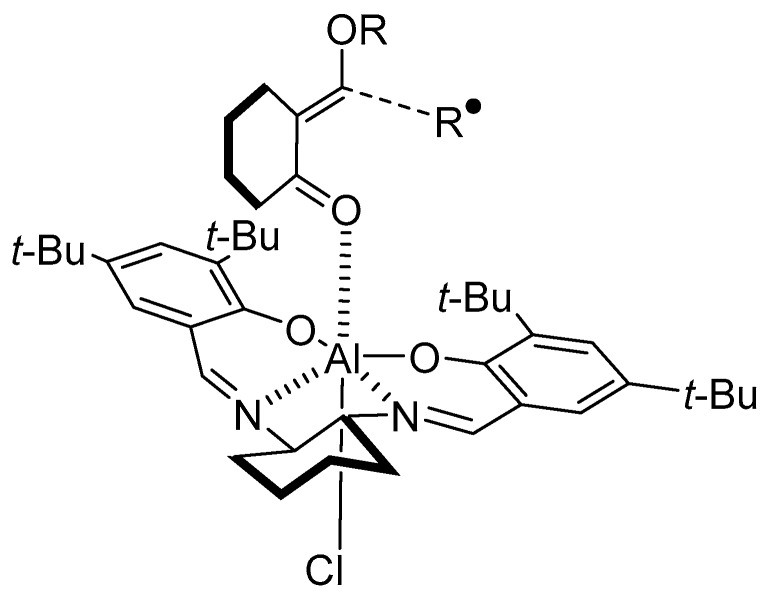
Proposed transition state in the addition of radicals to coordinating ketones.

An interesting use of Al(salen) was described by Sibi, who reported the radical addition to cyclic ketones, with a fixed *cis* geometry, performing the reaction in the presence of an Al(salen) complex (Scheme 10 and Figure 7) [57]. Stereoselective addition of radicals in Michael reactions is a challenging subject [58]. Recently, photoredox catalysis has been used to access the simple and accessible formation of radical precursors. Furthermore, stereoselective photoredox methodologies are under active investigations [59]. Suitable and available chiral Lewis acids, stable in reaction conditions and in the presence of radicals, are quite interesting for the above mentioned topic.

For the catalyst recovery in Michael reactions, the possibility to link the Al(salen) metal complexes to solid supports was examined. (*R*,*R*)-(Salen)AlCl complexes, immobilized on poly(norbornene)s, exhibited excellent activities and enantioselectivities as catalysts for the 1,4-conjugate addition of cyanide to α,β-unsaturated imides [60].

**Scheme 11:**
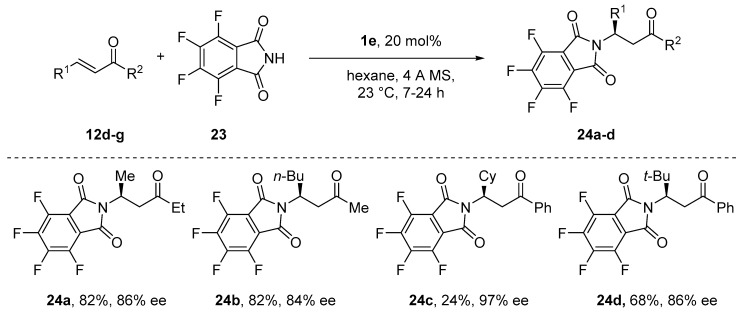
Asymmetric 1,4-addition of 3,4,5,6-tetrafluoro phthalimide to unsaturated ketones mediated by **1e**.

As was previously discussed, the imides showed an insufficient reactivity towards cyanides. Nevertheless, kinetic studies indicated that these polymer-supported catalysts were significantly more active than their corresponding unsupported analogues. Presumably, the catalyst is also able to play a role in activating the cyanide towards the addition. Recently, the salen μ-oxo [(*S*,*S*)-(salen)Al]_2_O **1e** was reported to catalyze the asymmetric 1,4-addition of 3,4,5,6-tetrafluoro- phthalimide **23** to unsaturated ketones (Scheme 11) [61].

The products are formed in up to 89% yield and 97% ee. The tetrafluoro phthalimide group can easily be removed under mild conditions to afford the free primary amines in high yields. The mechanistic analysis was carefully performed, considering kinetic and isotopic effects, and a study of non-linear effect. All the data suggested a dual activation mechanism. A pre-equilibrium formation of a 1:1 complex between tetrafluoro phthalimide and the catalyst was observed, and an Al(salen)-enone complex was formed. The rate-determining step is the addition of the tetrafluoro phthalimide catalyst complex to the catalyst-activated enone.

## 7. Reaction of Nucleophiles/Enolates Promoted by Chiral Al(salen) Complexes

**Scheme 12:**
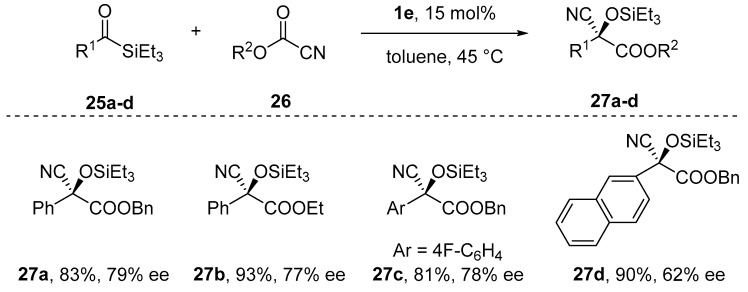
Use of Al(salen) metal complexes to achieve Brook rearrangement proposed by Johnson.

Chiral cyanohydrin derivatives can be easily accessed by Al(salen) catalysis. Cyanohydrins are masked nucleophiles and in the presence of strong bases, are converted to the corresponding anions able to react with suitable electrophiles. During the formation of the anionic cyanohydrin, stereochemical information is lost. For this reason, and due to the presence of a strong base, the reaction appears challenging. Johnson [62] has employed chiral Al(salen) metal complexes to generate the anion of protected cyanohydrins via a carbon-to-oxygen silyl migration (Brook rearrangement) and a subsequent reaction with an electrophile (Scheme 12).

After an extensive investigation of catalyst/metal alkoxide combination, *i*PrOAl(salen) catalyst was found to be the most effective catalyst. The reaction is quite sensitive to traces of water and it is carried out in toluene in sealed tube. Formation of the CNAl(salen), or its isomeric form NCAl(salen), was revealed. The nucleophilic cyanide then reacts with the acylsilane and a (metallo)silyloxynitrile is formed through the Brook rearrangement. This species is nucleophilic and reacts with the acylnitrile present in the reaction conditions to undergo an acylation reaction. In another reaction promoted by Al(salen), a nucleophilic species is formed under the reaction conditions, and is related to the nucleophilicity of isocyanide [63]: the classical Passerini [64] and Ugi [65] reactions. 

**Scheme 13:**
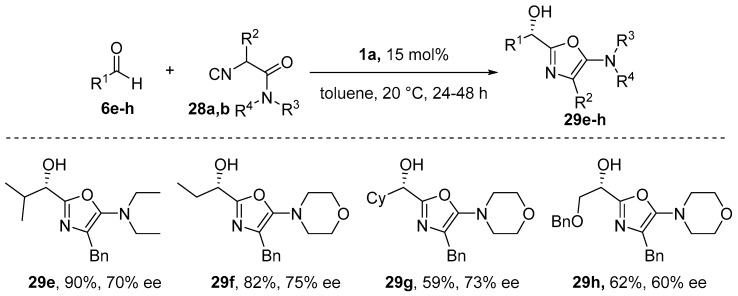
Enantioselective addition of α-isocyanoacetamides to aldehydes catalyzed by ClAl(salen).

These are well-known and powerful multicomponent reactions [66] that have found an extensive use in medicinal chemistry, synthesis, and catalysis. Multicomponent stereoselective Passerini reactions [67] were found quite difficult to develop, and only recently a stereoselective variant of the Ugi reaction [68] was reported. Quite remarkably, ClAl(salen) was found capable of catalyzing the enantioselective addition of α-isocyanoacetamides to aldehydes (Scheme 13) [69]. The catalyst tolerates a wide range of aldehydes to afford the diversely substituted 2-(1-hydroxyalkyl)-5-aminooxazoles **29** in good yields and enantioselectivities (Scheme 13).

**Figure 8:**
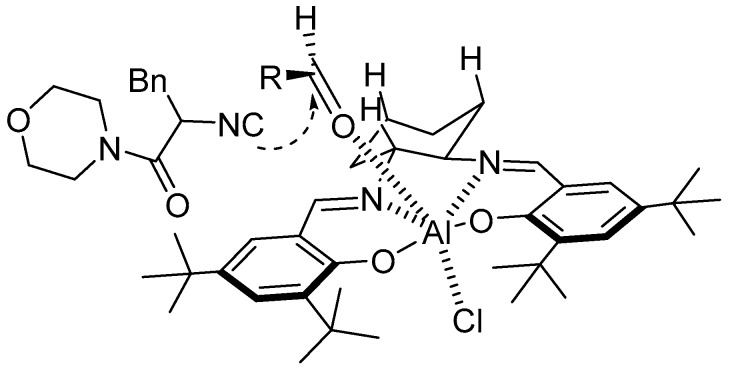
Proposed mechanism in the reaction of isocyanides with aldehydes.

The mechanism of the reaction considers that the Al(salen) behaves as a Lewis acid, activating the aldehyde towards the addition. As the (*S*)-configuration of the oxazole resulted from the addition reaction, the observed (*S*)-configured product most likely derives from an attack of the isocyanide nucleophile on the *Re*-face of the coordinated aldehyde (Figure 8).

The intermediate electrophile formed after the addition is captured by the nucleophilic oxygen of the amide moiety. Up to 80% ee was obtained, with the reaction showing a sufficient scope, as both aliphatic and aromatic aldehydes were found reactive.

## 8. Cycloaddition and Multicomponent Stereocontrolled Reactions Promoted by Chiral Al(Salen) Complexes

**Scheme 14:**
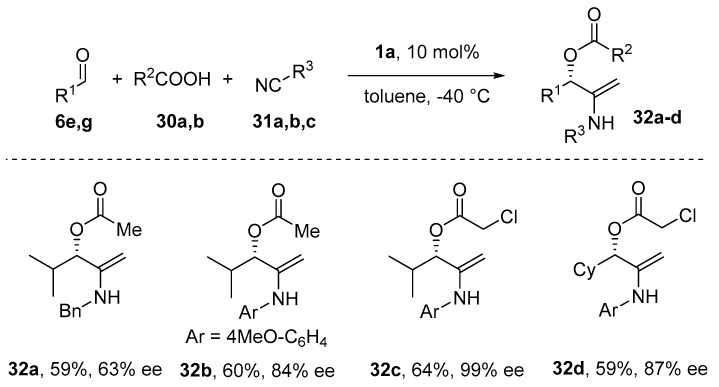
Passerini three-component reaction catalyzed by Al(salen) complexes.

As mentioned in the previous section, the development of a truly catalytic enantioselective three-component Passerini reaction was a challenge for a long time, due to some factors related to the intricate consecutive mechanism, and the rate of the uncatalyzed reaction. In addition, as the components are Lewis basic, the catalyst turnovers and its acidity need to be taken into account. Al(salen) complexes are rather stable in the presence of carboxylic acid, and they are able to promote a stereoselective variant of the Passerini reaction in the presence of isocyanide and aldehydes (Scheme 14) [70].

**Scheme 15:**
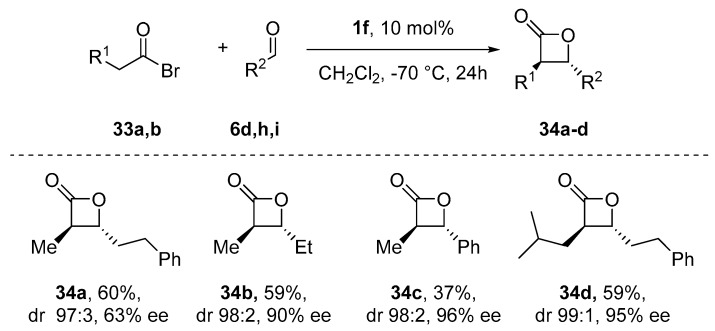
*trans*-Stereoselective catalytic formation of β-lactones by cyclocondensation with acyl bromides in the presence of Al(salen).

In the standardized protocol for the reaction, it is important to add slowly the carboxylic acid to minimize the background reaction. The stereoselectivity of the reaction was also influenced by the group coordinated to the Al(salen). According to the reported results, the presence of a Cl as an electron-withdrawing substituent on the Al center was crucial to improve the stereoselectivity of the reaction. Again, for this reaction, the use of Al(salen) was absolutely necessary as other salen metal complexes were found active, but gave a relatively modest stereoselection. To eliminate the background competing pathways, the reaction needs to be performed at −40 °C. The selectivity of the reaction improved with less-reactive aromatic isocyanides. However, the reaction was only reported with aliphatic aldehydes. Different carboxylic acids were tested, and the presence of functional groups (Cl, SH, OH, alkene) was tolerated. By replacing the carboxylic acid with hydrazoic acid, which we have already seen to be compatible with Al(salen) complexes, the same research group was able to perform a catalytic enantioselective synthesis of 5-(1-hydroxyalkyl)tetrazoles [71]. The reaction can be seen as a three-component Passerini reaction (P-3CR) of aldehyde, isocyanide and hydrazoic acid. The reaction is applicable to a wide range of aliphatic aldehydes and to both aromatic and aliphatic isocyanides with tetrazoles (45–99% yields, 51–95% ees). Peters has reported an Al(salen) *trans*-stereoselective catalytic formation of β-lactones by cyclocondensation with acyl bromides (Scheme 15) [72].

The Al(salen) catalyst bearing pyridinium rings and its efficiency were studied by inserting different substituents on the heteroaromatic residues. A more active and selective catalyst was obtained. The reaction can be rationalized by the in situ formation of a ketene that is transformed into the corresponding aminium enolate. The [2+2] cycloaddition occurs between the aldehyde coordinated to the aluminum Lewis acid, and the aminium enolate. The most effective Al(salen) complex is obtained by the reaction with AlMe_3_. Enantiomeric excesses up to 96% in favor of the *trans* diastereoisomer are obtained.

## 9. Chiral Al(salen) Complexes, CO_2_ and Related Electrophiles

**Scheme 16:**
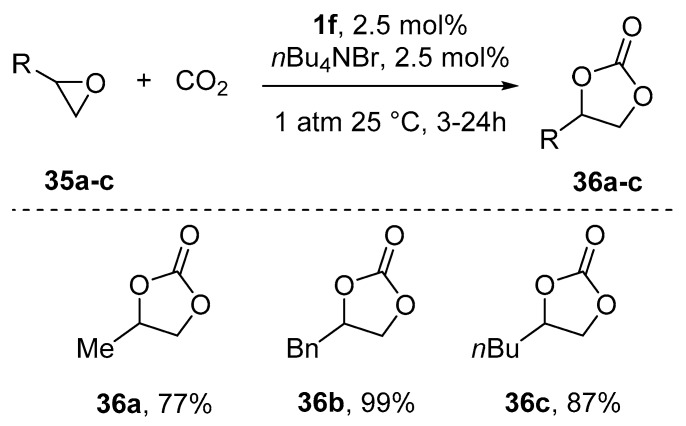
Synthesis of cyclic carbonates from epoxides catalyzed by a bimetallic oxo- aluminum(salen) complex.

The global warming that is causing severe climate problems is caused by the increasing levels of atmospheric carbon dioxide [73]. Controlling, reducing and using CO_2_ as natural source for chemicals will represent a formidable challenge for the near future. Under this perspective, the use of Al(salen) for CO_2_ activation and fixation has been explored. Chiral Al(salen) metal complexes have been reported in stereoselective reactions with CO_2_. In addition, other related electrophiles, such as CS_2_ or R-N=C=O have also been studied. One important reaction employed in many catalytic studies with CO_2_ is its insertion into epoxides to generate cyclic carbonates or a polycarbonate, interesting products from the industrial point of view [74]. Epoxides can be obtained from many sources. Many new green methodologies for their production were developed, with cheap oxidants and under mild conditions [75]. Many catalytic processes for the obtainment of cyclic carbonates from epoxides and CO_2_ have been reported, and some of them are based on Al(salen) complexes [76,77,78]. Al(salen) offers a number of advantages, such as the activity reported, and the possibility to use simple reaction conditions. However, in these preliminary studies, racemic Al(Salen) complexes were employed. Using all the experience gained in salen chemistry, North reported [79,80] an enantio-enriched chiral bimetallic oxo aluminum(salen) complex which showed relatively good activities for the synthesis of cyclic carbonates by using terminal epoxides at room temperature and pressure conditions (Scheme 16).

Various oxo Al(salen) derivatives were synthesized and tested in a model reaction using styrene oxide and carbon dioxide. The presence of tetrabutylammonium bromide as co-catalyst was mandatory, even at greater carbon dioxide pressures. The mechanism of the reaction was investigated in detail [81]. The second-order dependence of the reaction rate on tetrabutylammonium bromide concentration was observed and in addition, the authors detected the presence of tributylamine in the reaction mixture. Under the reaction conditions, tetrabutylammonium bromide decomposed to tributylamine. In the catalytic cycle the Al(salen) complex is acting as a Lewis acid coordinating the epoxide and activating it toward ring opening by the bromide nucleophile. The arising ammonium ion reacts reversibly with carbon dioxide to form carbamate salt, linked to Al(salen). This carbamate is in a preorganized arrangement for an intramolecular reaction with the bromo alkoxide derived from the epoxide. 

**Scheme 17:**
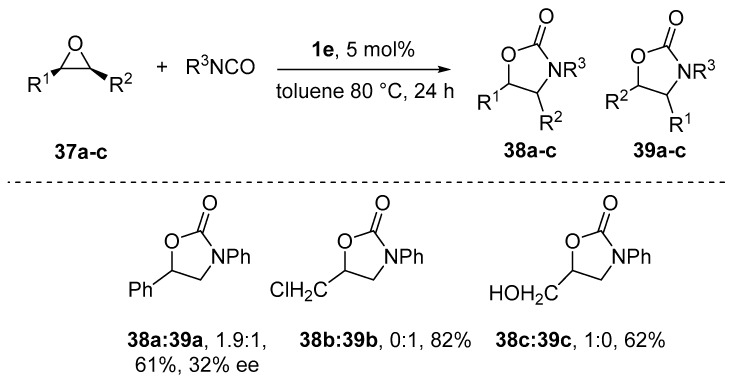
Synthesis of oxazolidinones promoted by catalytic amount of an Al(salen) complex.

Finally, the observed cyclic carbonate is obtained by intramolecular S_N_2 reaction of the Al alkoxide with the bromo substituted carbon. Optimization of the process for reducing its cost was also reported, and a practical methodology for the synthesis of the Al(salen) complex in situ, avoiding the use of expensive aluminum alkyl derivative, was reported [82]. The combined use of [Al(salen)]_2_O and tetrabutylammonium bromide (or tributylamine) was also reported to promote the addition of carbon disulfide to epoxides, to produce 1,3-oxathiolane-2-thiones [83]. North also reported the synthesis of oxazolidinones promoted by catalytic amount of Al(salen) complexes, in a reaction of epoxides and isocyanates (Scheme 17) [84].

The reaction proceeded with overall retention of epoxide stereochemistry, and both aromatic and aliphatic isocyanates were employed as reacting substrates. The reaction is performed in the absence of co-catalysts and the proposed mechanism considered the breaking and reforming of the Al−O−Al bridge as the reaction progressed. Finally, the reaction with carbon dioxide can be used for a practical and inexpensive methodology, the kinetic resolution [85] of terminal epoxides [86]. The reaction was carried out under mild conditions (0−25 °C and 1 bar of CO_2_ pressure) in the presence of tetrabutylammonium bromide as a co-catalyst and in the absence of solvent. Although the relative reaction rate of the two enantiomers of epoxide (k_rel_) was not high and substrate-dependent, the highest k_rel_ obtained was 15.4 and it was possible to obtain moderate enantiomeric excesses for the desired product.

## 10. Polymerization Reactions Promoted by Chiral Al(Salen) Complexes

**Scheme 18:**
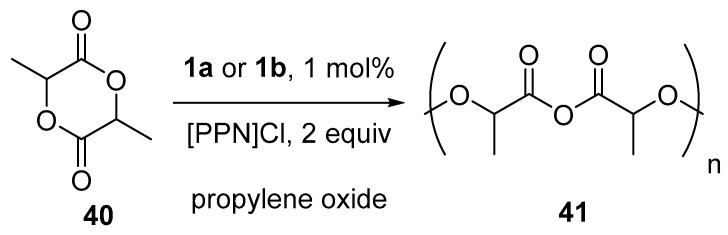
ROP of a rac-lactide catalyzed by an Al(salen) complex generated in situ.

Biodegradable and biocompatible polyesters are important polymers that have found a wide range of practical applications [87]. Among all the polyesters reported, polylactide (PLA), which is obtained from lactic acid as a renewable source, has recently assumed a wide importance due to its biodegradability and biocompatibility [88]. Polylactide is generally obtained via ring-opening polymerization (ROP) of lactides, promoted by organometallic compounds. Many metal alkoxides were proved to be suited for such polymerizations. Among all the metals employed, aluminum-based catalysts are quite efficient initiators, due to their ability to control the polymer tacticities by modification of the ancillary ligands [89,90,91,92,93]. Under this perspective, the employment of chiral Salen-type Schiff bases to control stereospecificity is important and Al(salen) complexes are employed in the polymerization of lactides. Pang reported the synthesis of Salen-type aluminum complexes bearing binaphthyl-imine derivatives [94,95]. An aluminum salen complex generated in situ was reported to display a high activity for the ROP of a racemic lactide at room temperature (Scheme 18) [96].

DFT studies performed on the catalytic process were able to illustrate that initiation and propagation proceeded via an external alkoxide attack on the coordinated monomer. Polylactide efficient preparation was recently reported with a novel trinuclear salen−aluminum complex. In this process, the direct ring-opening polymerization (ROP) of inexpensive racemic lactide (rac-LA) occurs with high activity and stereoselectivity [97].

## 11. Conclusions

The ability of Al(salen) to act as an effective Lewis acid in many beinteresting transformations was highlighted and summarized in this review. Al(salen) metal complexes are particularly effective in promoting Michael reactions, compared to other salen metal complexes. Furthermore, one of the most effective Al(salen) catalysts is the oxo dimer obtained by a controlled hydrolysis. The remarkable stability towards nucleophiles and water conditions of these species can be further exploited under affordable and friendly reaction conditions. The interesting luminescent properties of Al(salen) can be advantageously explored in many stereoselective catalytic reactions. The simple and convenient chemistry necessary to introduce functional groups in the salen framework add other possibilities to this interesting picture. Tailored ligands, able to display bifunctional behaviors, are easily accessible. Furthermore, the coordination step for the preparation of Al complexes is straightforward and makes use of commercially available reagents. All these advantages are available to interested researchers who want to explore the fascinating chemistry of the Al(salen) for further developments.
